# SPECT/CT Imaging: A Noninvasive Approach for Evaluating Serial Changes in Angiosome Foot Perfusion in Critical Limb Ischemia

**DOI:** 10.1089/wound.2018.0924

**Published:** 2020-01-24

**Authors:** Ting-Heng Chou, Said A. Atway, Adam J. Bobbey, Timur P. Sarac, Michael R. Go, Mitchel R. Stacy

**Affiliations:** ^1^ The Center for Regenerative Medicine, The Research Institute at Nationwide Children's Hospital, Columbus, Ohio.; ^2^ Department of Orthopaedics, The Ohio State University College of Medicine, Columbus, Ohio.; ^3^ Department of Radiology, Nationwide Children's Hospital, Columbus, Ohio.; ^4^ Department of Surgery, The Ohio State University College of Medicine, Columbus, Ohio.

**Keywords:** perfusion imaging, angiography, diabetes mellitus, critical limb ischemia, peripheral arterial disease, angiosome

## Abstract

**Objective:** To investigate the feasibility of serial radiotracer-based imaging as a noninvasive approach for quantifying volumetric changes in microvascular perfusion within angiosomes of the foot following lower extremity revascularization in the setting of critical limb ischemia (CLI).

**Approach:** A CLI patient with a nonhealing foot ulcer underwent single-photon emission computed tomography (SPECT)/computed tomography (CT) imaging of the feet before and after balloon angioplasty of the superficial femoral artery (SFA) and popliteal artery. SPECT/CT imaging was used to evaluate serial changes in angiosome perfusion, which was compared to quantitative changes in peripheral vascular anatomy and hemodynamics, as assessed by standard clinical tools that included digital subtraction angiography (DSA), ankle-brachial index (ABI), and toe-brachial index (TBI).

**Results:** Following revascularization, upstream quantitative improvements in stenosis of the SFA (pre: 35.4% to post: 11.9%) and popliteal artery (pre: 59.1% to post: 21.7%) shown by DSA were associated with downstream angiosome-dependent improvements in SPECT microvascular foot perfusion that ranged from 2% to 16%. ABI measurement was not possible due to extensive arterial calcification, while TBI values decreased from 0.26 to 0.16 following revascularization.

**Innovation:** This is the first study to demonstrate the feasibility of assessing noninvasive volumetric changes in angiosome foot perfusion in response to lower extremity revascularization in a patient with CLI by utilizing radiotracer-based imaging.

**Conclusion:** SPECT/CT imaging allows for quantification of serial perfusion changes within angiosomes containing nonhealing ulcers and provides physiological assessment that is complementary to conventional anatomical (DSA) and hemodynamic (ABI/TBI) measures in the evaluation of lower extremity revascularization.

**Figure:**
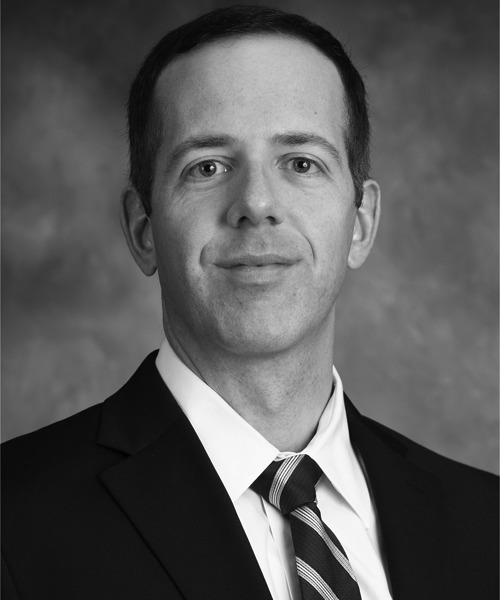
**Mitchel R. Stacy, PhD**

## Introduction

Peripheral arterial disease (PAD) is a progressive atherosclerotic disease of the lower extremities affecting more than 200 million people globally.^1^ Critical limb ischemia (CLI) represents the severe stage of PAD and is characterized by ischemic rest pain, nonhealing lower extremity ulcers, and/or gangrene that is attributable to significant arterial occlusive disease,^2,3^ significantly impacts patient quality of life, and is associated with high mortality and amputation rates.^3^ Therefore, prompt treatment as well as noninvasive tools that can effectively monitor treatment response and efficacy are critical for improving clinical outcomes in patients with CLI.

The primary treatment option for limb preservation in the setting of CLI is revascularization of the lower extremity. One specific revascularization approach that has emerged in recent years is angiosome-guided revascularization, which is based on the concept that angiosomes, or three-dimensional (3D) blocks of tissue supplied by specific upstream source arteries, can be utilized to guide targeting of specific downstream vascular runoff territories of the foot that contain nonhealing wounds, thereby improving rates of wound healing and limb salvage.^4–8^ While various noninvasive approaches have been utilized in the clinical setting to assess hemodynamic (ankle-brachial index [ABI] and toe-brachial index [TBI]) and anatomical (digital subtraction angiography [DSA] and x-ray computed tomography [CT] angiography) improvements associated with lower extremity revascularization, to date, no standard clinical tool exists for quantifying physiological improvements, such as tissue perfusion, within 3D angiosomes of the foot.

Our research team has recently developed a clinical imaging protocol that utilizes hybrid single-photon emission CT (SPECT)/CT imaging, CT-based image segmentation of angiosomes, and serial image registration, which allows for assessment of 3D angiosome foot perfusion under baseline conditions and has demonstrated potential for noninvasively detecting perfusion abnormalities in CLI patients with diabetes mellitus (DM).^9^ We hypothesized that this imaging technology and image processing approach could be further utilized to evaluate the response to revascularization in patients with CLI. Therefore, in the current study, we applied SPECT/CT imaging to assess the perfusion response to revascularization within specific 3D foot angiosomes in CLI patients with DM. In addition, our SPECT/CT perfusion results were compared to revascularization-induced changes in arterial stenosis, as assessed by conventional DSA, as well as serial changes in lower extremity hemodynamics, as evaluated by ABI and TBI.

## Clinical Problem Addressed

CLI patients often undergo revascularization procedures that are directed at improving downstream foot perfusion to facilitate wound healing and limb salvage. However, standard clinical tools, such as ABI, TBI, and Duplex ultrasound, generally provide information related to hemodynamic responses to revascularization, while other conventional approaches such as magnetic resonance (MR) angiography, x-ray CT angiography, and DSA, are limited to morphological changes in the peripheral atherosclerotic lesions. While noninvasive tools such as transcutaneous oxygen pressure (TcPO_2_) exist and provide some information related to microvascular perfusion of the foot, clinical techniques such as these are superficial in nature and do not allow for 3D assessment of angiosomes being targeted for revascularization. Therefore, in the present study, we present technological feasibility of utilizing SPECT/CT imaging to quantify serial physiological changes in 3D angiosome perfusion in a patient with DM and CLI who was scheduled to undergo lower extremity revascularization.

## Materials and Methods

A 63-year-old male patient (body mass index = 22.6; fasting glucose = 131 mg/dL) with CLI, type 2 DM, and nonhealing foot ulcers of the first and second digits of the right foot underwent SPECT/CT perfusion imaging before and after clinically indicated revascularization of the lower extremity. The patient was enrolled into a recently initiated prospective clinical trial (NCT03622359) focused on evaluating the utility of radiotracer-based imaging for the assessment of angiosome perfusion responses to lower extremity revascularization. The study protocol was approved by both the Institutional Review Board and Radiation Safety Committee and was in accordance with the guidelines set forth by the Declaration of Helsinki. All patients were provided written informed consent after receiving an explanation of the experimental procedures and potential risks associated with participating in the study.

Perfusion imaging was performed using a conventional hybrid SPECT/16-slice CT imaging system with large field-of-view sodium iodide detectors (Discovery NM/CT 670; GE Healthcare, Buckinghamshire, United Kingdom) before and after balloon angioplasty of the right superficial femoral artery (SFA) and popliteal artery. The patient received a low-dose intravenous radiotracer injection (14.6 mCi of ^99m^Tc-tetrofosmin) under resting conditions and underwent SPECT/CT imaging 20 min postinjection. Images were acquired at the level of the ankle and foot. The patient then returned 4 days following revascularization to undergo the same SPECT/CT imaging protocol. SPECT perfusion images were acquired using a 360° step and shoot acquisition with a 140.5 keV ±10% window, 3° projections, and 30 s per stop. Immediately after the SPECT acquisition, CT images were acquired for attenuation correction at a slice thickness of 0.625 mm, at 140 kVp, and 15 mA. All SPECT images were reconstructed using iterative reconstruction, applying corrections for attenuation and resolution loss (Xeleris; GE Healthcare).

**Figure 1.**
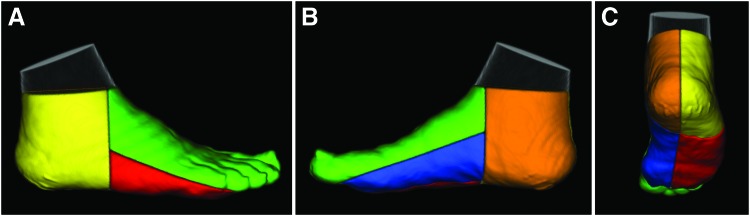
CT-based image segmentation approach for regional evaluation of angiosome foot perfusion. Volume rendering of the medial heel (*orange*), lateral heel (*yellow*), dorsal foot (*green*), medial plantar (*blue*), and lateral plantar (*red*) angiosomes overlaid on CT images, which are shown in the **(A)** lateral, **(B)** medial, and **(C)** posterior/plantar views. CT, computed tomography. Color images are available online.

**Figure 2.**
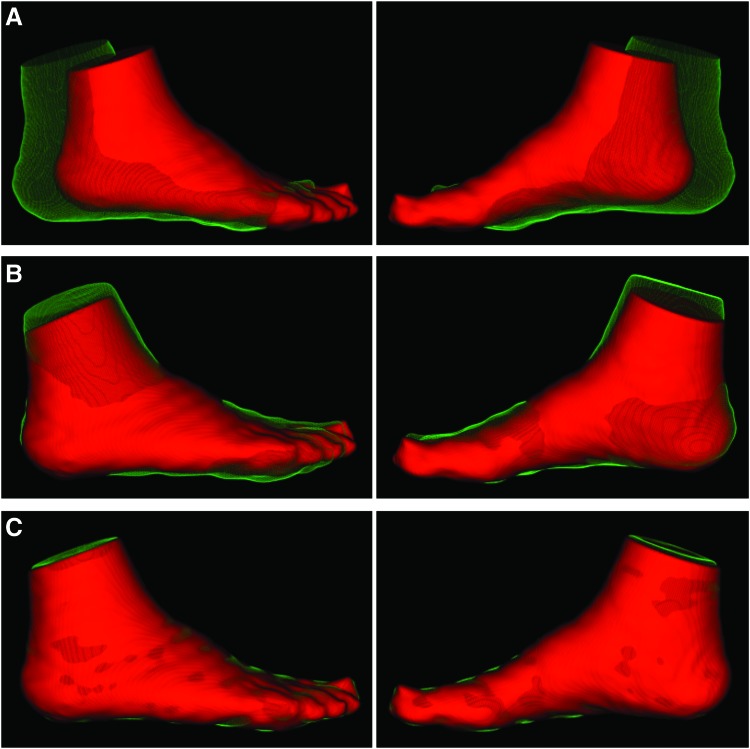
Serial image registration of CT images for evaluating serial changes in angiosome foot perfusion. Lateral and medial views of volume rendered CT images are displayed for the **(A)** initial foot position at both study time points before image registration, **(B)** following global rigid registration, and **(C)** after nonrigid registration. The *red images* represent the foot position at the time of patient's pre-revascularization study visit, while the *green images* represent the change in foot position at the time of patient's follow-up post-revascularization visit. Color images are available online.

Reconstructed SPECT images were coregistered with CT attenuation images, which were used to segment and define angiosomes of the feet (lateral plantar, medial plantar, lateral heel, medial heel, and dorsal foot; Fig. 1) using a previously developed image analysis tool kit (BioImage Suite) that our team has previously utilized for evaluating regional skeletal muscle perfusion ^9,10^ and oxygenation.^11^ Average radiotracer uptake was assessed from coregistered SPECT images within the CT-defined 3D angiosomes of the foot. Average SPECT image intensity values were normalized to injected radiotracer dose (mCi) and patient body weight (kg) to generate standardized uptake values (SUVs). Following evaluation of SPECT SUVs for the pre-revascularization time point, serial CT images from the pre- to post-revascularization time points were coregistered (Fig. 2) using a previously published automated technique that allowed for alignment of serial images.^9,11^ The relative percent change in SUV within each angiosome was calculated from the pre- to post-revascularization time point.

DSA images were acquired as part of standard of care during the revascularization procedure. Quantitative angiographic analysis was performed on the same arterial segments before and after revascularization procedures using a validated quantitative angiographic system (QAngio XA; Medis Medical Imaging Systems, The Netherlands). The minimal luminal diameter (MLD) and the mean reference diameter (RD), obtained from averaging a 5-mm segment proximal to the atherosclerotic lesion, were used for calculations of percent diameter stenosis [(1-MLD/RD) × 100]. Mean segment diameter represented the mean diameter of the treated arterial segment. The gain in mean segment diameter was defined as the change in mean segment diameter from baseline to the final postprocedural angiogram.

Pressure waveforms as well as the ABI and TBI were evaluated at both the pre- and post-revascularization time points to assess hemodynamic changes that may occur in the lower extremity after treatment. Specifically, serial systolic blood pressures were acquired on the left and right extremities for the brachial artery, dorsalis pedis artery, posterior tibial artery, and the great toes using Doppler ultrasound.

## Results

**Figure 3.**
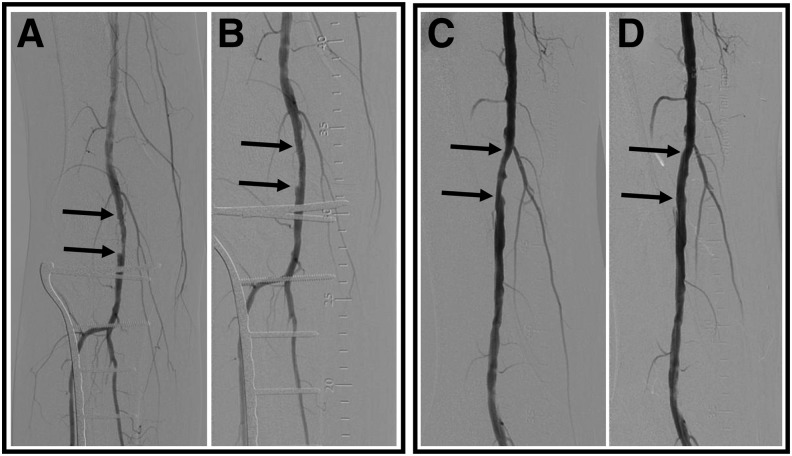
Digital subtraction angiography in a patient with CLI undergoing multivessel revascularization of the lower extremity. Angiographic images acquired **(A)** before and **(B)** after balloon angioplasty of the right popliteal artery, and **(C)** before and **(D)** after the balloon angioplasty of the right SFA demonstrate improvement in arterial patency following revascularization. *Arrows* denote segments of arterial stenosis targeted for balloon angioplasty. CLI, critical limb ischemia; SFA, superficial femoral artery.

All angiographic results from pre- to post-revascularization are summarized in Table 1. The patient presented with stenosis of the right popliteal artery (59.2% stenosis; lesion length = 25.8 mm) and the right SFA (35.42% stenosis; lesion length = 27.12 mm; Fig. 3). The patient underwent balloon angioplasty with a 5 × 40 mm drug-coated balloon for the popliteal artery and a 6 × 60 mm drug-coated balloon for the SFA. Following the revascularization procedures, percent stenosis of the popliteal artery improved from 59.2% to 21.8%, while percent stenosis of the SFA improved from 35.4% to 11.98%. The gains in mean lesion diameter were 1.71 mm (popliteal) and 1.17 mm (SFA), respectively, which could be visualized on DSA images (Fig. 3).

**Table 1.** Multimodality vascular assessment

	Vessels Treated	
*Angiography Analysis*		
Before revascularization	*SFA*	*POP*
Arterial stenosis (%)	35.42	59.16
MLD (mm)	3.25	1.84
Lesion length (mm)	27.12	25.81
Mean segment diameter (mm)	4.75	3.78
After revascularization		
Arterial stenosis (%)	11.98	21.76
MLD (mm)	4.42	3.55
Lesion length (mm)	7.15	18.47
Mean segment diameter (mm)	5.08	4.23
Lesion gains		
MLD gain (mm)	1.17	1.71
Mean segment diameter gain (mm)	0.33	0.45

	Pre	Post
*Hemodynamic Analysis*		
Ankle-brachial index	Undetected	Undetected
Toe-brachial index	0.26	0.16
Toe pressure (mmHg)	27	30

	Change in Perfusion (%)	
*SPECT/CT Image Analysis*		
Dorsal foot	2.56	
Medial plantar	16.45	
Medial heel	12.07	
Lateral plantar	2.43	
Lateral heel	3.61	

CT, computed tomography; MLD, minimal luminal diameter; POP, popliteal artery; SFA, superficial femoral artery; SPECT, single-photon emission computed tomography; SUV, standard uptake value.

**Figure 4.**
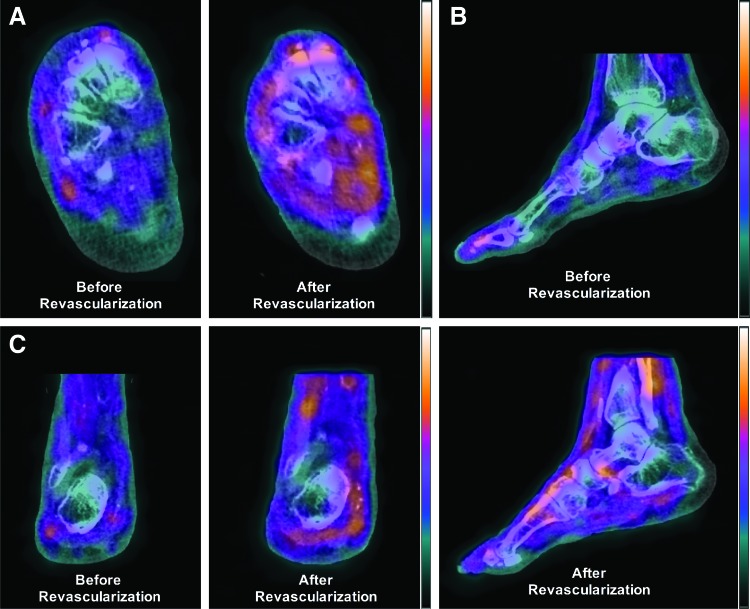
SPECT/CT perfusion imaging in a CLI patient before and after revascularization. Coregistered and fused SPECT/CT images demonstrate increased radiotracer uptake and improved microvascular perfusion in the foot in the **(A)** axial, **(B)** sagittal, and **(C)** coronal views following balloon angioplasty of the SFA and popliteal artery. SPECT, single-photon emission computed tomography. Color images are available online.

All angiosome perfusion results are summarized in Table 1. Following revascularization, angiosome perfusion in the ulcerated dorsal foot region increased by 2.56% (Fig. 4). In addition, similar perfusion changes were quantified in the lateral plantar (2.43%) and lateral heel (3.61%) angiosomes, while larger perfusion increases were quantified for the medial plantar (16.45%) and medial heel (12.07%) angiosomes.

The ABI was not assessable due to diffuse calcification of the lower extremities that resulted in noncompressible arteries. The absolute systolic pressure measured in the great toe of the right foot slightly increased from a baseline value of 27 mmHg to a post-revascularization value of 30 mmHg, while the baseline TBI value of 0.26 changed to 0.16 after revascularization (Table 1).

## Discussion

In the present report, we have demonstrated for the first time the utility of SPECT/CT imaging for quantitative assessment of serial changes in microvascular perfusion within specific 3D angiosomes of the foot following revascularization. In addition to quantifying heterogeneous perfusion responses in the foot, we demonstrated that these changes in perfusion were associated with angioplasty-induced quantitative improvements in upstream arterial dimensions, but not in agreement with traditional hemodynamic measures of the foot, thus supporting the notion that SPECT/CT perfusion imaging may offer complementary novel insight into physiological alterations that occur in specific vascular territories of the foot following revascularization.

While a variety of noninvasive tools have been utilized to diagnose and evaluate PAD and CLI in the clinical setting, most have been limited to assessment of hemodynamics or vascular anatomy. TcPO_2_ is the only widely utilized tool in the clinical setting for assessing foot perfusion; however, TcPO_2_ is superficial in nature, does not have a large field-of-view, and often does not allow for assessment of the plantar surface of the foot. In addition, while MR-based imaging approaches such as contrast-enhanced imaging,^12^ arterial spin labeling,^13,14^ and blood oxygen level dependent^11,15^ can quantify volumetric tissue oxygenation and perfusion of the lower extremities, these approaches are not capable of measuring perfusion under resting conditions and generally require pharmacological, reactive hyperemia, or exercise stress paradigms. Our radiotracer-based imaging approach with SPECT/CT imaging has previously demonstrated utility for quantifying regional volumetric perfusion under resting conditions in both the preclinical^10^ and clinical^9^ setting and offers a fast and straightforward imaging protocol for assessing limb perfusion in a patient population at high risk for lower extremity ulceration and amputation. In addition, SPECT/CT imaging does not require the use of intravenous iodinated contrast agents and instead utilizes ^99m^Tc-tetrofosmin, a relatively safe agent that has no known contraindications, thus offering a safe technique for evaluating lower extremity perfusion in severe PAD and CLI patients who commonly present with impaired renal function.^16,17^ In the present case, we have demonstrated through the use of our image segmentation and registration approach, the further utility of SPECT/CT imaging for quantifying serial changes in perfusion within specific 3D angiosomes of the foot after revascularization in a CLI patient with nonhealing wounds.

Quantitative analysis of DSA images that were acquired before and after balloon angioplasty of the SFA and popliteal artery demonstrated treatment-induced improvements in upstream arterial diameter that were associated with subsequent downstream improvements in angiosome perfusion quantified by SPECT/CT imaging. While increased diameters of the upstream SFA and popliteal artery should theoretically result in diffuse improvements in foot perfusion that are angiosome independent, SPECT/CT imaging revealed heterogeneous improvements in foot perfusion that were angiosome dependent. Thus, the poor perfusion responses to revascularization that were quantified by SPECT/CT imaging for certain angiosomes may be due to additional downstream minor occlusions in arteries, microvasculature, or a combination of the two. For example, the present patient in this report demonstrated higher perfusion responses (12% and 16%) within the medial plantar and medial heel angiosomes, which may suggest that the supplying posterior tibial and medial plantar arteries may be absent of significant atherosclerosis. Alternatively, perfusion in the lateral plantar, lateral heel, and dorsal foot angiosomes increased by ∼2–3%, which may suggest that the supplying anterior tibial and peroneal arteries, as well as the microvasculature in these specific angiosomes, could possess varying degrees of atherosclerosis that are undetectable by conventional DSA. Therefore, in the present case, SPECT/CT perfusion imaging appears to offer complementary evaluation of regional physiological responses to revascularization that could be used in combination with conventional anatomical imaging.

In addition to DSA, standard ABI and TBI measurements were attempted and acquired for comparison to SPECT/CT perfusion imaging results. However, due to diffuse arterial calcification in the present patient, ABI measurements were not possible, which is a common occurrence and problem with ABI measurements for many patients with DM. Also, while absolute measures of systolic blood pressure in the great toe of the revascularized foot did improve from 27 to 30 mmHg, TBI measurements actually decreased, further highlighting common inconsistencies and limitations of hemodynamic measurements in the setting of CLI and DM. Thus, while ABI and TBI are frequently used in the clinical evaluation of PAD patients, SPECT/CT perfusion imaging may offer additional functional assessment for angiosomes being specifically targeted for revascularization.

In summary, this report demonstrates the potential utility of SPECT/CT imaging for quantifying revascularization-induced changes in microvascular perfusion within specific 3D angiosomes of the foot, which could complement standard clinical tools such as DSA, ABI, and TBI. Additional clinical studies are warranted to investigate the utility of SPECT/CT perfusion imaging as a noninvasive tool for evaluating the efficacy of the treatment in PAD and CLI patients.

## Innovation

This is the first reported case to demonstrate the feasibility of assessing noninvasive volumetric changes in angiosome foot perfusion in response to lower extremity revascularization by utilizing radiotracer-based imaging. In addition, we show that our SPECT/CT perfusion imaging approach is complementary to conventional techniques for anatomical evaluation (DSA), and may offer further insight into the physiological changes in microvascular perfusion that occur within specific vascular territories of the foot that are being targeted for revascularization. Further application of SPECT/CT imaging in PAD patients may offer a novel noninvasive approach for evaluating perfusion response to revascularization and assist with guiding medical treatment.

Key Findings

- SPECT/CT imaging allows for serial assessment of the perfusion response to peripheral angioplasty within the 3D angiosome of the foot that contains a nonhealing ulcer and is being targeted for revascularization.
- SPECT/CT-derived changes in angiosome foot perfusion following revascularization are associated with upstream improvements in arterial diameter.
- SPECT/CT perfusion imaging provides 3D regional assessment of the perfusion responses to medical treatment, which could offer a more sensitive technique for evaluating local changes in tissue physiology than standard hemodynamic measures such as ABI and TBI.

## About the Authors

**Ting-Heng Chou, PhD,** is a postdoctoral scientist in the Center for Regenerative Medicine at the Research Institute at Nationwide Children's Hospital, where his research focuses on nuclear imaging of PAD in the clinical and preclinical settings. **Said A. Atway, DPM,** is a podiatrist in the Department of Orthopedics at the Ohio State University College of Medicine who specializes in the treatment of chronic lower extremity wounds. **Adam J. Bobbey, MD,** is a nuclear medicine physician and chief of the nuclear medicine division in the Department of Radiology at Nationwide Children's Hospital. **Timur P. Sarac, MD,** is a Professor of Surgery and serves as the chief of vascular surgery at the Ohio State University College of Medicine. **Michael R. Go, MD,** is an Associate Professor and serves as the Director of Ohio State's Limb Preservation Program. **Mitchel R. Stacy, PhD,** is a Principal Investigator in the Center for Regenerative Medicine at the Research Institute at Nationwide Children's Hospital and an Assistant Professor in the Department of Surgery at the Ohio State University College of Medicine. He serves as the principal investigator of an ongoing NIH supported clinical trial focused on radiotracer-based imaging for evaluation of perfusion responses to revascularization in patients with critical limb ischemia.

## Abbreviations and Acronyms

3D: three-dimensional
ABI: ankle-brachial index
CLI: critical limb ischemia
CT: computed tomography
DM: diabetes mellitus
DSA: digital subtraction angiography
MLD: minimal luminal diameter
MR: magnetic resonance
PAD: peripheral arterial disease
RD: reference diameter
SFA: superficial femoral artery
SPECT: single-photon emission computed tomography
SUV: standardized uptake values
TBI: toe-brachial index
TcPO_2_: transcutaneous oxygen pressure
